# Circular RNA circ_0081001 knockdown enhances methotrexate sensitivity in osteosarcoma cells by regulating miR-494-3p/TGM2 axis

**DOI:** 10.1186/s13018-020-02169-5

**Published:** 2021-01-13

**Authors:** Wei Wei, Liefeng Ji, Wanli Duan, Jiang Zhu

**Affiliations:** Department of Orthopedics, Shaoxing Shangyu People’s Hospital, No. 517 Civic Avenue, Baiguan Street, Shangyu District, Shaoxing, Zhejiang, 312300 China

**Keywords:** circ_0081001, miR-494-3p, TGM2, MTX sensitivity

## Abstract

**Background:**

Circular RNAs (circRNAs) have been shown to participate in the chemoresistance and tumorigenesis of multiple cancers. The purpose of this research was to investigate the function of circ_0081001 in methotrexate (MTX) resistance of osteosarcoma (OS) and its potential molecular mechanism.

**Methods:**

The expression of circ_0081001, cytochrome P450 family 51 subfamily A member 1 (CYP51A1), and miR-494-3p was detected by qRT-PCR. Cell viability, apoptosis, migration, and invasion were evaluated by Cell Counting Kit-8 (CCK-8) assay, flow cytometry, and transwell assay, respectively. Western blot (WB) assay was used to measure the protein levels of cleaved-caspase3 (cleaved-casp3), E-cadherin, N-cadherin, and transglutaminase-2 (TGM2). The interaction between miR-494-3p and circ_0081001 or TGM2 was predicted by bioinformatics analysis and verified using the dual-luciferase reporter assay. The mice xenograft model was established to investigate the roles of circ_0081001 in MTX resistance of OS in vivo.

**Results:**

Circ_0081001 and TGM2 were upregulated, and miR-494-3p was downregulated in MTX-resistant OS tissues and cells. Moreover, circ_0081001 interference enhanced cell sensitivity to MTX through promoting apoptosis and inhibiting cell viability and metastasis in vitro. Furthermore, circ_0081001 was identified as a molecular sponge of miR-494-3p to upregulate TGM2 level. In addition, circ_0081001 knockdown inhibited MTX resistance via upregulating miR-494-3p and downregulating TGM2. Besides, circ_0081001 downregulation improved MTX sensitivity of OS in vivo.

**Conclusion:**

Knockdown of circ_0081001 enhanced MTX sensitivity of OS cells through downregulating TGM2 by sponging miR-494-3p, elucidating a novel regulatory mechanism for chemoresistance of OS and providing a potential circRNA-targeted therapy for OS.

## Highlights

- Circ_0081001 and TGM2 were overexpressed and miR-494-3p was lowly expressed in MTX-resistant OS tissues and cells.
- Circ_0081001 modulated TGM2 expression via acting as a molecular sponge of miR-494-3p in MTX-resistant OS cells.
- Knockdown of circ_0081001 inhibited MTX resistance in OS cells by upregulation of miR-494-3p and downregulation of TGM2.

## Introduction

Osteosarcoma (OS) is the most frequent primary malignant bone cancer and the second leading cause of cancer-related deaths in children and adolescents [1]. Over the past few decades, despite great efforts have been made to diagnose and treat OS, the prognosis remains very poor because of tumor recurrence, metastases, and chemoresistance [2, 3]. Moreover, the subtypes of OS are correlated with histologic response to chemotherapy and the histologic response to chemotherapy is significantly correlated with prognosis [4]. Methotrexate (MTX)-based chemotherapy remains a common strategy for treatment of OS, while tumor recurrence frequently occurs after development of MTX resistance [5]. Thus, seeking novel therapeutic targets and understanding the molecular mechanism of MTX resistance are important for OS treatment.

Circular RNAs (circRNAs), a new type of non-coding RNAs, are closed-loop structures formed by reverse splicing without 3′-end and 5′-end (unlike lncRNA) [6]. Increasing evidence has proven that circRNAs are more stable and difficult to degrade in response to the RNA exonuclease due to their closed-loop structures [7]. It has been suggested that several circRNAs play critical roles in pathogenesis of OS, such as modulating cancer cell proliferation, cell cycle, and chemoresistance, acting as prognostic biomarkers in patients with OS [8, 9]. Circ_0081001 (chr7:91755566-91756945) is derived from back-splicing of cytochrome P450 family 51 subfamily A member 1 (CYP51A1) transcript and has been suggested to serve as a possible biomarker for OS diagnosis and prognosis [10]. However, the exact function and regulatory mechanism of circ_0081001 in the progression of OS and MTX sensitivity remain largely unknown.

CircRNAs commonly exert their biological roles via serving as microRNA (miRNA) sponges in various cancers [11]. MicroRNAs (miRNAs) play essential roles in diseases through interaction with mRNAs [12]. MiR-494-3p has been reported to function as an anti-oncogene or oncogene in different cancers, as a tumor promoter in glioma [13] and hepatocellular carcinoma [14], as a tumor inhibitor in prostate cancer [15] and breast cancer [16]. Moreover, it has been reported that miR-494 played an anti-cancer role in OS [17]. In addition, miR-494 has been reported to be associated with chemoresistance in many cancers [18, 19]. However, the effect of miR-494-3p on MTX sensitivity in OS has not been reported.

Transglutaminase 2 (TGM2), a member of the transglutaminase family, has been identified to play essential roles in cell growth, apoptosis, metastasis, and chemosensitivity [20, 21]. Besides, previous report revealed that TGM2 promoted the progression of osteosarcoma [22]. Moreover, TGM2 was also reported to be related to chemoresistance in multiple cancers [23, 24]. Nevertheless, there is no evidence that TGM2 plays a role and the relationship between TGM2 and miR-494-3p in MTX sensitivity of OS. Interestingly, online bioinformatics database showed that circ_0081001 and miR-494-3p had complementary binding sequence for miR-494-3p, which prompted us to assume the ceRNA network of circ_0081001/miR-494-3p/TGM2.

In this research, we explored the roles of circ_0081001, miR-494-3p, and TGM2 in MTX sensitivity of OS. Additionally, we investigated the circ_0081001/miR-494-3p/TGM2 regulatory network in MTX-resistant OS cells. We aimed to provide a new insight and treatment strategy for OS drug resistance.

## Materials and methods

Tissue samples (*n* = 63) were collected from OS patients who received MTX treatment at Shaoxing Shangyu People’s Hospital. The patients with OS were divided into the sensitive (*n* = 35) group and resistant (*n* = 28) group according to the Response Evaluation Criteria In Solid Tumors (RECIST) [25]. The tissues were timely frozen in liquid nitrogen and preserved in – 80 °C until use. In this study, every patient provided written informed consent. And the research was authorized by the Research Ethics Committee of Shaoxing Shangyu People’s Hospital.

### Cell culture and transfection

OS cell lines (U2OS and HOS) were bought from ATCC (Manassas, VA, USA). These cells were cultivated in DMEM (Invitrogen, Waltham, MA, USA) containing 10% fetal bovine serum (FBS; Invitrogen) at 37 °C in a humidified atmosphere with 5% CO_2_. Continuous exposure to gradually increasing concentrations of MTX (Lingnan, Guangdong, China) was used to establish MTX-resistant U2OS and HOS cells (U2OS/R and HOS/R). The cells were incubated at an initial concentration of MTX (0.1 μg/mL) for 24 h, and then the medium was changed. When the cells grew well, cells were exposed to MTX (0.1 μg/mL) for 24 h, and then repeated the above steps 7 times. Next, the surviving cells were exposed to increasing doses of MTX (0.25, 0.5, 1, 5, 10, and 20 μg/mL). After that, cells were cultured with MTX (5 μg/mL) to maintain the resistance.

Short hairpin RNA (shRNA) interference targeting circ_0081001 (sh-circ_0081001) and corresponding control (sh-NC), miR-494-3p mimic (miR-494-3p) and corresponding control (miR-NC), miR-494-3p inhibitor (anti-miR-494-3p) and corresponding control (anti-miR-NC), TGM2 overexpression vector (TGM2), and corresponding control (vector) were bought from Genepharma (Shanghai, China). U2OS/R and HOS/R cells were transfected with oligonucleotides (50 nM) or vectors (2 μg) using Lipofectamine 3000 reagent (Invitrogen).

### Quantitative real-time polymerase chain reaction (qRT-PCR)

Total RNA was isolated by Trizol reagent (Invitrogen). The RNA samples were reversely transcribed to complementary DNA (cDNA) using the TIANScript RT Kit (Tiangen Biotech, Beijing, China). The expression of circ_0081001, CYP51A1, and miR-494-3p was examined by qRT-PCR analysis using the SYBR green PCR kit (TaKaRa, Dalian, China) and TaqMan miRNA assay (Applied Biosystems, Carlsbad, CA, USA) on an ABI 7900 system (Applied Biosystems). The expression of each gene evaluated using the 2^−ΔΔCt^ method, followed by normalizing to GAPDH or U6. The primers were as follows: circ_0081001, sense: 5′-CATGCAGCCTGGCTCTTACC-3′, antisense: 5′-CTGCTCCAAGAAAACCTGAAACT-3′; CYP51A1, sense: 5′-CAGAACTCCTCAGACTGTGG-3′, reverse: 5′-GTCTTTGATTGACAGTGGGA-3′; miR-494-3p, sense: 5′-GCTCCGTGAACCAACTCG-3′, antisense: 5′-GGGTGAAACACACACGGGAA-3′; GAPDH, sense: 5′-AATGGGCAGCCGTTAGGAAA-3′, antisense: 5′-GAAGGGGTCATTGATGGCA-3′; U6, sense: 5′-CTCGCTTCGGCAGCACATATACT-3′, antisense: 5′-ACGCTTCACGAATTTGCGTGTC-3′.

### RNase R treatment

In order to evaluate the stability of circ_0081001 and CYP51A1 (its linear isoform), total RNA (2 μg) was incubated in RNase R (3 U/μg, Epicentre Technologies, Madison, WI, USA) at 37 °C for 30 min. After that, the cells were harvested and subsequently subjected to qRT-PCR to examine the abundance of circ_0081001 and CYP51A1.

### Subcellular fractionation location

Cytosolic and nuclear fractions were isolated with PARIS Kit (Life Technologies Corp., Grand Island, NY, USA). In short, U2OS/R and HOS/R cells were collected and were gently washed with ice-cold PBS twice. Afterward, the cells were re-suspended in fractionation buffer. After centrifugation, the cytoplasmic fraction was acquired from the supernatant content, whereas the remaining nuclear pellet was again lysed using the cell disruption buffer as nuclear fraction. At last, the enrichment of circ_0081001, GAPDH, and U6 in the nuclear and cytoplasmic fractions was determined by qRT-PCR. GAPDH served as control for cytoplasmic and U6 functioned as control for nuclear.

### Cell viability assay

Cell Counting Kit-8 (CCK-8; Beyotime, Shanghai, China) was utilized for detecting cell viability. In brief, U2OS/R and HOS/R cells were placed into a 96-well plate. CCK-8 (10 μL) reagent was added after transfection, followed by incubation for 2–3 h. The absorbance was examined under a microplate reader at 450 nm. MTX concentration causing 50% inhibition of growth (IC50) was measured using the relative survival curve.

### Cell apoptosis assay

According to the recommendations, Annexin V-FITC/PI apoptosis detection kit (Sangon Biotech, Shanghai, China) was applied for measuring cell apoptosis. Briefly, U2OS/R and HOS/R cells were collected and stained with Annexin V-FITC and PI in the darkness for 15 min. Finally, flow cytometry (Partec AG, Arlesheim, Switzerland) was applied to detect the number of apoptotic cells.

### Transwell assay

Twenty-four-well transwell chamber (8 μm pores, Corning Incorporation, Corning, NY, USA) was coated with Matrigel (BD Biosciences) to determine cell invasion capacity and uncoated membrane was employed to detect cell migration. In brief, cells re-suspended in FBS-free medium (200 μL) were seeded in top chamber. Medium with FBS (500 μL) in the bottom chamber was used as chemoattractant. Non-migrated and non-invaded cells on the top surfaces were carefully wiped off by a cotton wool after 24 h of incubation. Paraformaldehyde (4%) was used to fix cells that invaded or migrated to the lower surface of the chamber, followed by staining with crystal violet (0.1%). Lastly, an inverted microscope was applied to count and photograph the migrated and invaded cells.

### Western blot (WB) assay

Total protein was extracted using RIPA lysis buffer (Applygen, Beijing, China) containing phenylmethanesulfonyl fluoride (Beyotime), followed by quantitation with the BCA protein assay kit (Applygen). Then, protein (25 μg) from each sample was resolved by SDS-PAGE, followed by being transferred to polyvinylidene difluoride membranes (Beyotime). The membranes were incubated for 12 h at 4 °C by primary antibody against cleaved-caspase3 (cleaved-casp3) (1:500, ab2302; Abcam, Cambridge, UK), E-cadherin (1:800, ab15148; Abcam), N-cadherin (1:800, ab18203; Abcam), TGM2 (1:2000, ab137378; Abcam), and GAPDH (1:3000, ab70699; Abcam) after blocking with 5% non-fat dried milk. After that, the secondary antibody (1:5000, D110058; Sangon Biotech) was used to incubate the membranes for 2 h. Finally, the ECL kit (Applygen) was employed to visualize the protein bands. Protein levels were normalized to GAPDH and evaluated using ImageJ software.

### Dual-luciferase reporter assay

Online software (circinteractome and starbase) was used to predict the potential target genes of circ_0081001 and miR-494-3p. Fragment from circ_0081001 and TGM2 3′UTR that contained wild-type (WT) or mutant (MUT) binding sites for miR-494-3p was synthesized and then inserted into pmirGlO luciferase reporter vector (Promega, Madison, WI, USA) to generate WT plasmids (WT-circ_0081001 and TGM2 3′UTR WT) or MUT plasmids (MUT-circ_0081001 and TGM2 3′UTR MUT). U2OS/R and HOS/R cells were co-transfected with plasmid (WT or MUT) and miR-494-3p (or miR-NC). Following cultivation for 48 h, dual-luciferase assay system (Promega) was applied to evaluate luciferase activity.

### Xenograft mice model

Animal experiments were granted by committee of Animal Research of Shaoxing Shangyu People’s Hospital. U2OS/R cells were transfected with sh-NC or sh-circ_0081001. Next, stably transfected cells (1 × 10^6^) were subcutaneously injected into the left flank of the BALB/c nude mice (male, 6-week-old, *n* = 6/group, Shanghai Experimental Animal Center, Shanghai, China) and treated with MTX twice a week. Tumor volume was evaluated every week using slide calipers and calculated using the formula: 0.5 × length × width^2^. Mice were sacrificed after 4 weeks, and tumors were excised, weighed, and collected for detecting circ_0081001, miR-494-3p, and TGM2.

### Statistical analysis

In our research, the data were presented as the mean ± standard deviation (SD). Each experiment was repeated at least three times. Statistical analyses were performed by GraphPad Prism 6.0. The statistical differences were estimated by Student’s *t* test (two groups) or one-way analysis of variance (ANOVA; mutiple groups). Receiver operating characteristic (ROC) curves were established by MedCalc version 11.4.2.0 software (MedCalc Software bvba, Ostend, Belgium) to distinguish between patients with OS who responded to MTX and those who did not respond to MTX. Kaplan–Meier method was applied to generate the survival curve. *P* < 0.05 was regarded as statistically significant.

## Results

### Circ_0081001 was overexpressed in MTX-resistant OS tissues and cells, and positively correlated with poor prognosis

**Fig. 1:**
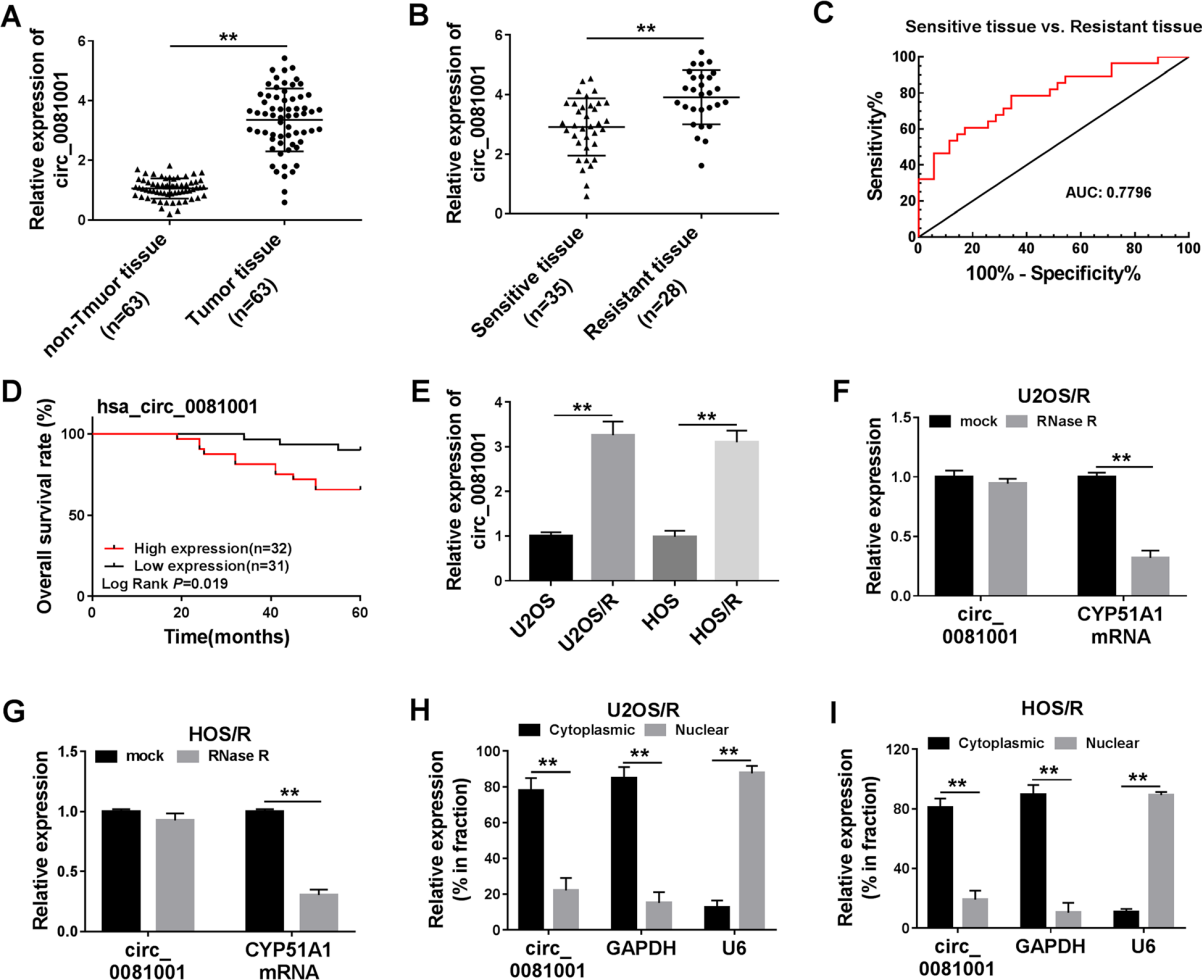
Circ_0081001 was overexpressed in the OS tissues and cell lines. **a** The expression of circ_0081001 was measured in non-tumor and tumor tissues by qRT-PCR. **b** The expression of circ_0081001 was determined in patients responding or not responding to MTX treatment by qRT-PCR. **c** ROC curve analysis of circ_0081001 expression was used to distinguish sensitive tissues from resistant tissues. **d** The overall survival rate was analyzed between low and high circ_0081001 expression group in OS patients by Kaplan-Meier survival analysis. **e** Relative expression of circ_0081001 was evaluated by qRT-PCR in U2OS, U2OS/R, HOS, and HOS/R cells. **f**, **g** The relative levels of circ_0081001 and CYP51A1 mRNA were examined by qRT-PCR after treatment of RNase R in U2OS/R and HOS/R cells. **h**, **i** The qRT-PCR assay was applied to determine the subcellular location of circ_00081001 in U2OS/R and HOS/R cells. **P < 0.01

The qRT-PCR was conducted to investigate circ_0081001 expression in OS tissues. It was found that circ_0081001 expression was upregulated in OS tissues (*n* = 63) compared with those in non-tumor tissues (*n* = 63) (Fig. 1a). According to RECIST, the patients were divided into sensitive (35 patients) and resistant (28 patients) groups. The data indicated that the expression of circ_0081001 was higher in patients who did not respond to treatment with MTX than those who responded to MTX (Fig. 1b). Additionally, we analyzed the diagnostic potential of circ_0081001 through conducting ROC analysis. The results proved that circ_0081001 had a good value in distinguishing sensitive tissue from resistant tissue with an area under curve (AUC) of 0.7796 (95% confidence interval, 0.6642–0.8949) (Fig. 1c). Moreover, 63 patients were divided into high expression hsa_circ_0081001 (*n* = 32) group or low hsa_circ_0081001 (*n* = 31) expression group based on median value of hsa_circ_0081001 expression. Patients with high hsa_circ_0081001 expression group showed poor survival in contrast to low expression group (*P* = 0.019) (Fig. 1d). Similarly, the abundance of circ_0081001 was also enhanced in U2OS/R and HOS/R cells relative to in U2OS and HOS cells (Fig. 1e). The qRT-PCR results showed that circ_0081001 was resistant to RNase R compared with the linear CYP5A1 (Fig. 1f, g), disclosing that circ_0081001 had a loop structure. Next, we examined the localization of circ_0081001 abundance in U2OS/R and HOS/R cells. The results presented that circ_0081001 was mainly located in the cytoplasm (Fig. 1h, i). These findings implied that circ_0081001 might have crucial roles in progression and chemotherapy of OS.

### Circ_0081001 knockdown enhanced MTX sensitivity by reducing cell viability and metastasis and increasing apoptosis in MTX-resistant OS cells

**Fig. 2:**
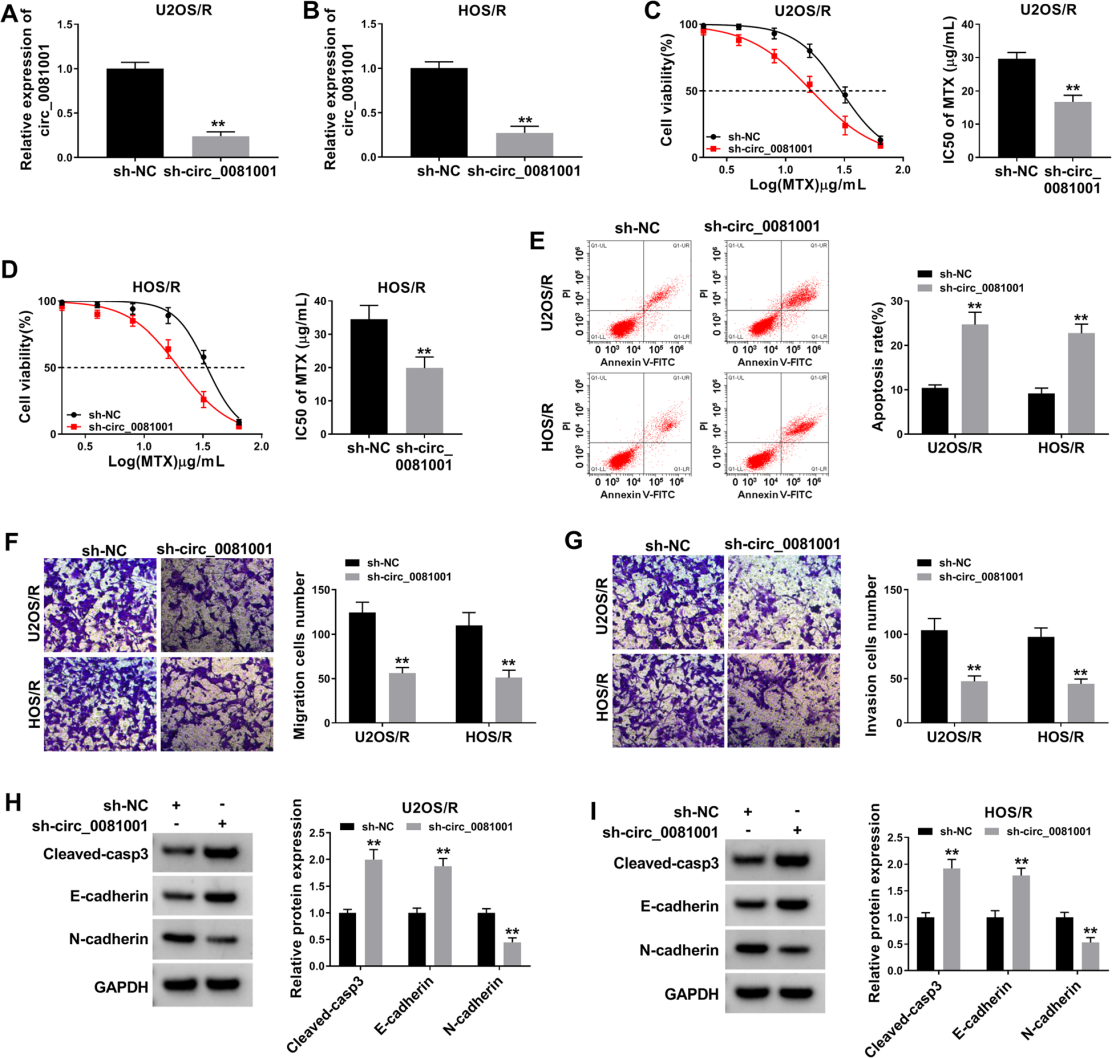
Downregulation of circ_0081001 inhibited MTX resistance by reducing cell viability and metastasis and promoting cell apoptosis in MTX-resistant OS cells. **a**, **b** The expression of circ_0081001 was analyzed in U2OS/R and HOS/R cells after transfection with sh-NC or sh-circ_00081001 using qRT-PCR analysis. **c**, **d** Cell viability and IC50 value were assessed by CCK-8 assay in U2OS/R and HOS/R cells transfected with sh-NC or sh-circ_00081001 and exposed to different concentrations of MTX. **e** Flow cytometry was performed to determine the cell apoptosis in U2OS/R and HOS/R cells after transfection with sh-NC or sh-circ_00081001. **f**, **g** Migration and invasion of U2OS/R and HOS/R cells were evaluated using the transwell assay after transfection with sh-NC or sh-circ_00081001. **h**, **i** WB analysis was conducted to examine the protein levels of cleaved-casp3, E-cadherin, and N-cadherin in U2OS/R and HOS/R cells after transfection with sh-NC or sh-circ_00081001. **P < 0.01

To explore the effect of circ_0081001 on MTX resistance, MTX-resistant OS cells were transfected with sh-circ_0081001 or sh-NC. The results proved that circ_0081001 level was obviously declined in U2OS/R and HOS/R cells transfected with sh-circ_0081001 in comparison with sh-NC group (Fig. 2a, b), implying that transfection efficiency of sh-circ_0081001 was relatively high. CCK-8 analysis showed that knockdown of circ_0081001 led to significant reduction of cell viability and decrease of MTX IC50 value in U2OS/R and HOS/R cells (Fig. 2c, d). Flow cytometry analysis indicated that the apoptotic rate was remarkably enhanced in U2OS/R and HOS/R cells after transfection of sh-circ_0081001 (Fig. 2e). Transwell assay indicated that circ_0081001 interference effectively inhibited migration and invasion of U2OS/R and HOS/R cells (Fig. 2f, g). Besides, WB assay was carried out to examine the protein abundance of cleaved-casp3 (a key executor in apoptotic process), E-cadherin (an epithelial marker), and N-cadherin (a mesenchymal marker). We found that the knockdown of circ_0081001 increased the protein expression of cleaved-casp3 and E-cadherin while decreased the protein abundance of N-cadherin in U2OS/R and HOS/R cells (Fig. 2h, i). Altogether, these results suggested that circ_0081001 downregulation sensitized U2OS/R and HOS/R cells to MTX.

### MiR-494-3p was a direct target of circ_0081001 and its expression was downregulated in MTX-resistant OS tissues and cells

**Fig. 3:**
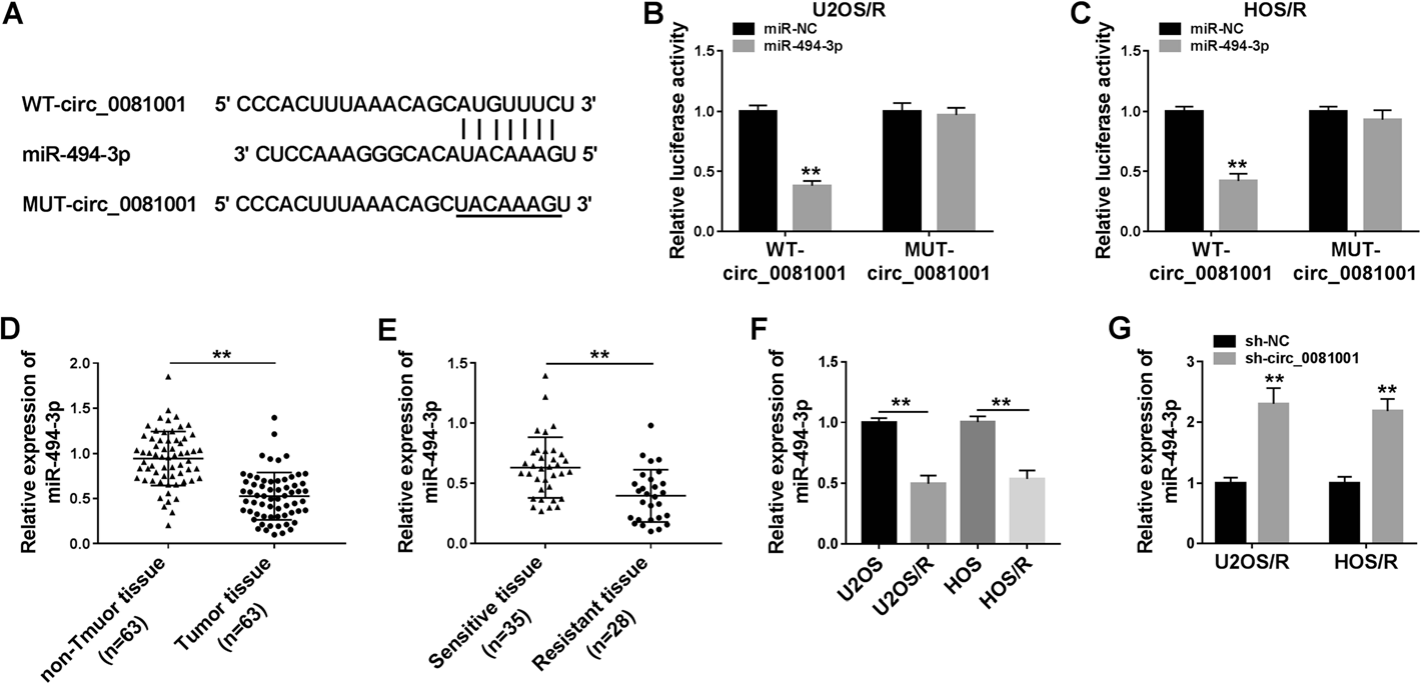
Circ_0081001 directly interacted with miR-494-3p in MTX-resistant OS cells. **a** The complementary binding sequence of miR-494-3p and circ_00081001 was predicted by circinteractome. **b**, **c** Relative luciferase activity was detected in U2OS/R and HOS/R cells after co-transfection with WT-circ_00081001 or MUT-circ_00081001 and miR-494-3p or miR-NC. **d** The abundance of miR-494-3p was evaluated by qRT-PCR in non-tumor and tumor tissues. **e** The level of miR-494-3p was examined by qRT-PCR in sensitive tissues and resistant tissues. **f** The expression of miR-494-3p was measured by qRT-PCR in U2OS, U2OS/R, HOS, and HOS/R cells. **g** QRT-PCR was conducted to detect the expression of miR-494-3p in U2OS/R and OS/R cells transfected with sh-NC or sh-circ_00081001. **P < 0.01

To explore whether circ_0081001 could act as miRNA sponge in MTX-resistant OS cells, online software circinteractome was used to predict target miRNAs. As presented in Fig. 3a, circ_0081001 contained possible binding sites of miR-494-3p. The dual-luciferase reporter assay was conducted to assess whether miR-494-3p could bind to circ_0081001. The luciferase activity of WT-circ_0081001 was significantly decreased after transfection with miR-494-3p in U2OS/R and HOS/R cells, but the luciferase activity of MUT-circ_0081001 was not greatly influenced after overexpression of miR-494-3p (Fig. 3b, c). After that, we analyzed the expression of miR-494-3p in tissues (tumor, non-tumor, sensitive, and resistant tissues) and cells (U2OS, HOS, U2OS/R, and HOS/R). As depicted in Fig. 3d, miR-494-3p abundance was reduced in tumor tissues relative to non-tumor tissues. And we also found that miR-494-3p level was lowly expressed in resistant tissues in contrast to sensitive tissues (Fig. 3e). Likewise, miR-494-3p level was declined in MTX-resistant cells (U2OS/R and HOS/R) cells relative to parental cells (U2OS and HOS) (Fig. 3f), and we found that knockdown of circ_0081001 increased miR-494-3p expression in U2OS/R and HOS/R cells (Fig. 3g). Collectively, these results demonstrated that circ_0081001 could directly interact with miR-494-3p.

### TGM2 was a downstream target of miR-494-3p

**Fig. 4:**
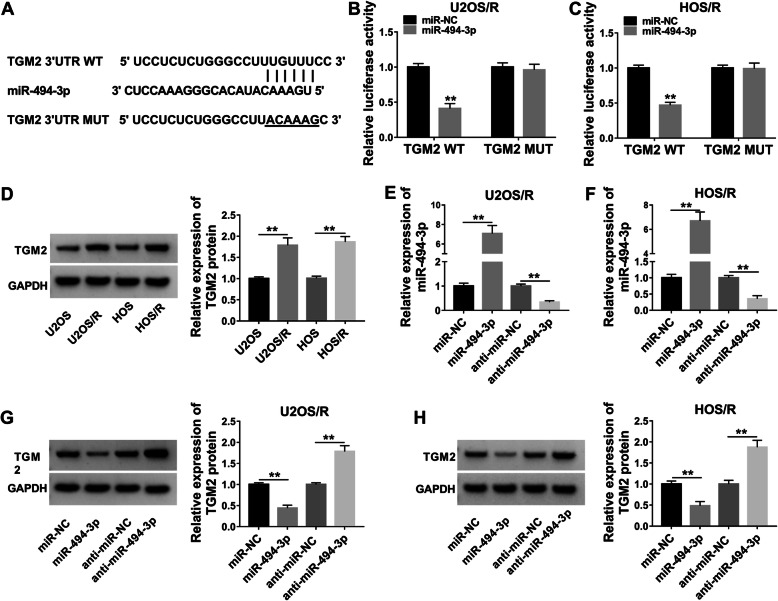
TGM2 was a direct target of miR-494-3p in MTX-resistant OS cells. **a** Starbase showed that miR-494-3p potentially targeted TGM2. **b**, **c** Dual-luciferase reporter assay was conducted to test the luciferase activity in U2OS/R and HOS/R cells co-transfected with TGM2 WT or TGM2 MUT and miR-494-3p or miR-NC. **d** WB assay was performed to analyze the protein expression of TGM2 in U2OS, U2OS/R, HOS, and HOS/R cells. **e**, **f** Relative miR-494-3p expression was detected using the qRT-PCR analysis in U2OS/R and HOS/R cells after transfection with miR-NC, miR-494-3p, anti-miR-NC, or anti-miR-494-3p. **g**, **h** The protein abundance of TGM2 was assessed by WB analysis in U2OS/R and HOS/R cells after transfection with miR-NC, miR-494-3p, anti-miR-NC, or anti-miR-494-3p. **P < 0.01

MiRNAs exert their functions through modulating expression of their downstream target genes [26]. Thus, online software starBase was employed to predict target genes of miR-494-3p. As presented in Fig. 4a, TGM2 had several binding sites within miR-494-3p. Transfection of miR-494-3p apparently reduced the luciferase activity of TGM2 WT, but miR-494-3p mimic had no impact on the luciferase activity of TGM2 MUT in U2OS/R and HOS/R cells (Fig. 4b, c). Moreover, we observed that the protein abundance of TGM2 was higher in MTX-resistant cells (U2OS/R and HOS/R) than in parental cells  (U2OS and HOS) (Fig. 4d). We used qRT-PCR analysis to detect transfection efficiency. As presented in Fig. 4e, f, miR-494-3p expression was elevated after transfection with miR-494-3p in U2OS/R and HOS/R cells, while transfection of anti-miR-494-3p showed an opposite effect. Next, we investigated the impact of miR-494-3p on TGM2 expression. Furthermore, miR-494-3p accumulation markedly reduced the protein abundance of TGM2, and downregulation of miR-494-3p drastically increased the protein abundance of TGM2 in U2OS/R and HOS/R cells (Fig. 4g, h). Our findings proved that miR-494-3p could bind with TGM2.

### Circ_0081001 regulated TGM2 expression through sponging miR-494-3p in MTX-resistant OS cells

**Fig. 5:**
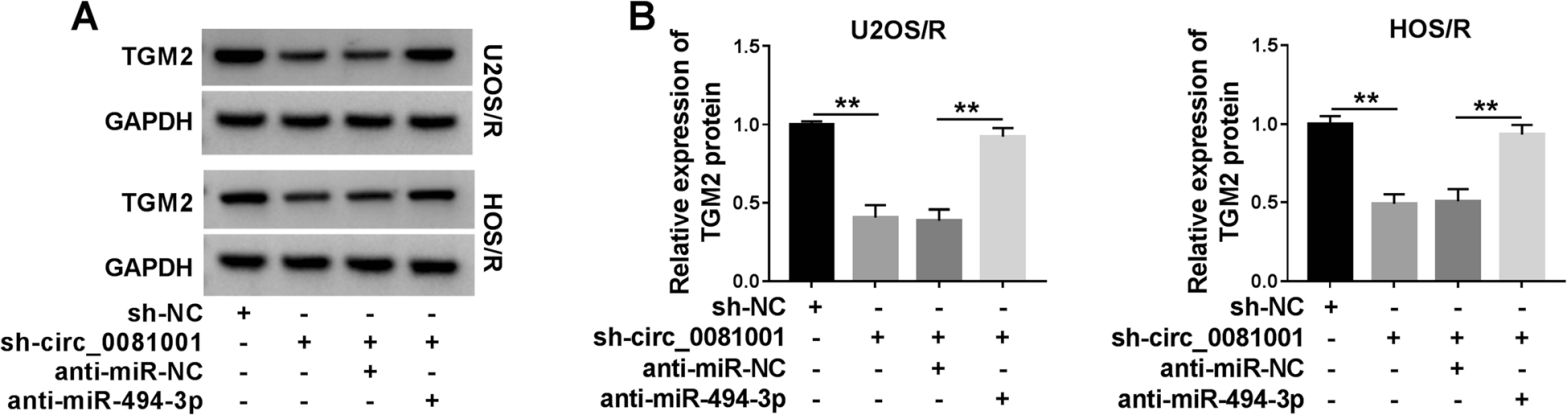
The expression of TGM2 was regulated by circ_00081001 and miR-494-3p in MTX-resistant OS cells. **a**, **b** WB assay was employed to examine the protein level of TGM2 in U2OS/R and HOS/R cells after transfection with sh-NC, sh-circ_00081001, sh-circ_00081001 + anti-miR-NC, or sh-circ_00081001 + anti-miR-494-3p. **P < 0.01

To probe whether circ_0081001 functioned as a sponge of miR-494-3p to modulate TGM2 expression, U2OS/R and HOS/R cells were transfected with sh-NC, sh-circ_0081001, sh-circ_0081001 + anti-miR-NC, or sh-circ_0081001 + anti-miR-494-3p. Knockdown of sh-circ_0081001 led to a decrease of TGM2 protein expression, while the effect was abated by downregulating miR-494-3p (Fig. 5a, b). Thus, these results proved that circ_0081001 sponged miR-494-3p to regulate TGM2 expression.

### Interference of circ_0081001 increased MTX sensitivity by upregulating miR-494-3p and downregulating TGM2 in MTX-resistant OS cells

**Fig. 6.**
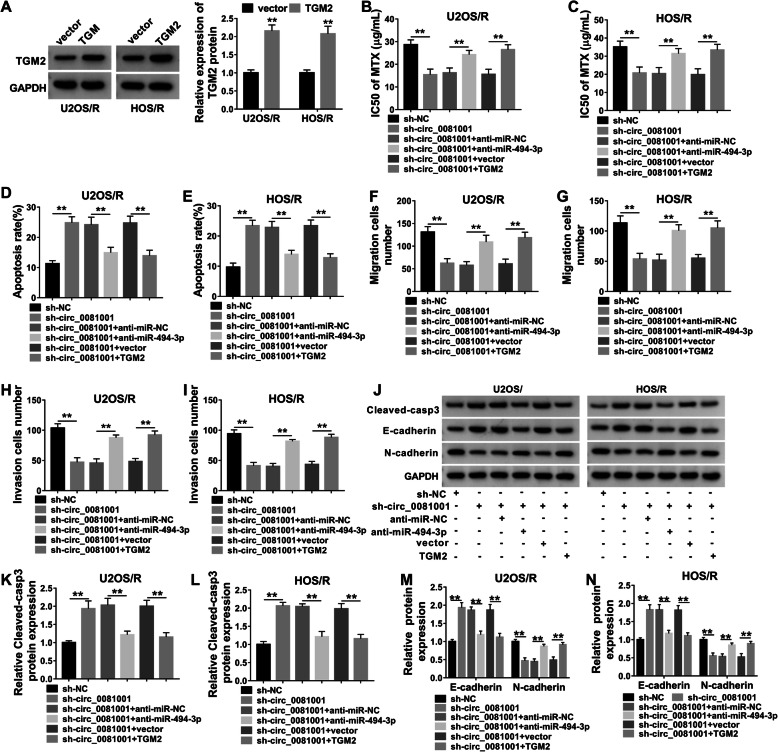
Knockdown of circ_0081001 enhanced MTX sensitivity through regulating miR-494-3p and TGM2 expression in MTX-resistant OS cells. **a** The protein abundance of TGM2 was measured using WB analysis in U2OS/R and HOS/R cells after transfection with vector and TGM2. **b**–**n** U2OS/R and HOS/R cells were transfected with sh-NC, sh-circ_0081001, sh-circ_0081001 + anti-miR-NC, sh-circ_0081001 + anti-miR-494-3p, sh-circ_0081001 + vector, or sh-circ_0081001 + TGM2. **b**, **c** IC50 value for MTX was calculated in MTX-treated U2OS/R and HOS/R cells. **d**, **e** Cell apoptosis was measured by flow cytometry. **f**–**i** Transwell assay was utilized to test cell migration and invasion capacities. **j**–**n** The protein levels of cleaved-casp3, E-cadherin and N-cadherin were determined by WB analysis. **P < 0.01

WB was used to examine the transfection efficiency. We found that the protein abundance of TGM2 was increased in U2OS/R and HOS/R cells after transfection with TGM2 (Fig. 6a), suggesting transfection of TGM2 was successful. We next analyzed whether the potential role of circ_0081001 in MTX-resistant OS cells was mediated by miR-494-3p and TGM2, U2OS/R and HOS/R cells were transfected with sh-NC, sh-circ_0081001, sh-circ_0081001 + anti-miR-NC, sh-circ_0081001 + anti-miR-494-3p, sh-circ_0081001 + vector, or sh-circ_0081001 + TGM2. CCK-8 analysis revealed that IC50 value of MTX was reduced in MTX-treated U2OS/R and HOS/R cells after transfection with sh-circ_0081001, while the effect was reversed by downregulating miR-494-3p or upregulating TGM2 (Fig. 6b, c). Interference of miR-494-3p or overexpression of TGM2 attenuated sh-circ_0081001-induced apoptosis in U2OS/R and HOS/R cells (Fig. 6d, e). Meanwhile, the suppressive effects of sh-circ_0081001 deficiency on migration and invasion were relieved by decreasing miR-494-3p or enhancing TGM2 (Fig. 6f–i). Also, miR-494-3p knockdown or TGM2 upregulation partially abolished the effects of circ_0081001 interference on promotion of cleaved-casp3 and E-cadherin expression as well as inhibition of N-cadherin abundance in U2OS/R and HOS/R cells (Fig. 6j–n). Therefore, these results revealed that circ_0081001 modulated MTX sensitivity through regulating miR-494-3p/TGM2 axis.

### Knockdown of circ_0081001 and treatment of MTX inhibited tumor growth via regulating miR-494-3p/TGM2 axis

**Fig. 7:**
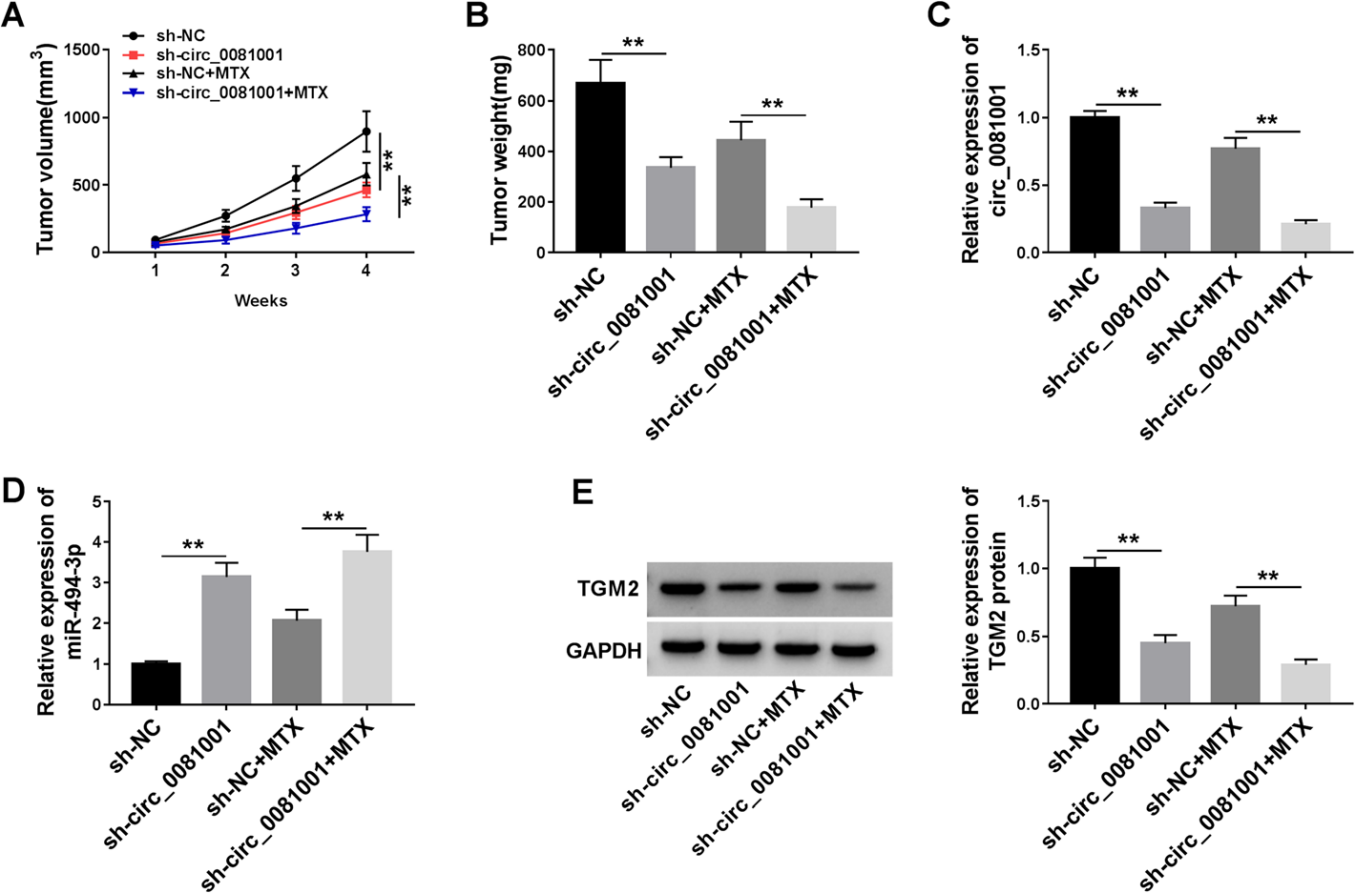
Deficiency of circ_0081001 elevated MTX sensitivity of OS in vivo. U2OS/R cells transfected with sh-NC or sh circ_0081001 were inoculated subcutaneously into the nude mice. The mice were treated with MTX (5 mg/kg MTX) twice a week after injection for 1 week. **a**, **b** Tumor volume and weight were examined. **c**, **d** The expression of circ_0081001 and miR-494-3p was measured by qRT-PCR in resected tumor tissues. **e** The protein abundance of TGM2 was detected by WB assay in resected tumor masses. **P < 0.01

To confirm the biological function of circ_0081001 and its underlying molecular mechanism in OS in vivo, U2OS/R cells with stable knockdown or control cells were subcutaneously injected into nude mice and treated with or without MTX twice a week. In agreement with in vitro data, knockdown of circ_0081001 decreased tumor volume and weight, and combination of sh-circ_0081001 and MTX treatment conspicuously inhibited tumor growth compared with only MTX treatment group (Fig. 7a, b). Additionally, interference of circ_0081001 declined the expression of circ_0081001 and TGM2 while promoted the expression of miR-494-3p in resected tumor tissues (Figs. 7c and 6e). Meanwhile, circ_0081001 knockdown together with MTX treatment obviously repressed the expression of circ_0081001 and TGM2, as well as enhanced the abundance of miR-494-3p in contrast to only MTX treatment group (Figs. 7c and 6e). The above results showed that circ_0081001 knockdown could enhance MTX sensitivity by upregulation of miR-494-3p and downregulation of TGM2 in vivo.

## Discussion

Although MTX-based chemotherapy is especially effective in a variety of cancers, chemoresistance development has become a major obstacle in cancer treatment [27]. Numerous studies have demonstrated that circRNAs play pivotal roles in modulating tumor processes and chemoresistance [28, 29]. In the present research, we focused on the functional role and underlying mechanism of circ_0081001 in MTX resistance in OS.

Emerging evidence has demonstrated that circRNAs are abundant in the human transcriptome and recognized as significant prognostic biomarkers in various cancers [6, 30]. Besides, emerging evidence demonstrated that dysregulation of circRNAs was tightly related to chemoresistance in OS. For instance, Yan et al. reported that circPVT1 knockdown contributed to cisplatin sensitivity in lung cancer cells by miR-181a-5p-mediated autophagy [31]. Zhang et al*.* found that circ_001569 facilitated cisplatin resistance in OS through activating of Wnt/β-catenin signaling pathway [32]. However, the effects of circ_0081001 on MTX resistance in OS have not been reported. In this research, we observed that circ_0081001 expression was upregulated in OS tissues, which was in line with previous study [10]. Additionally, we found that circ_0081001 expression also enhanced in MTX-resistant OS tissues and cells, suggesting that circ_0081001 dysregulation might be associated with MTX resistance in OS. Functional analysis further revealed that interference of circ_0081001 increased MTX sensitivity by reducing cell viability and metastasis as well as inducing apoptosis in MTX-resistant OS cells. These data proved that circ_0081001 conferred MTX resistance in MTX-resistant OS cells in vitro.

Recently, many investigations have focused on the interaction between circRNAs and microRNAs, and the importance of this interaction in chemoresistance [33]. One popular hypothesis indicates that circRNAs may serve as miRNA sponges to modulate their downstream targets [34]. Then, circinteractome tool was applied to predict the targets of circ_0081001, the results displayed that miR-494-3p might be a target of circ_0081001. Dual-luciferase reporter assay demonstrated that circ_0081001 directly interacted with miR-494-3p. Multiple miRNAs are abnormally expressed in cancers and may play critical roles in drug resistance [35, 36]. MiR-494 has been suggested to be lowly expressed in OS and its restoration represses OS cell growth and metastasis by suppressing insulin receptor substrate-1 [37]. Nevertheless, the influence of miR-494-3p on MTX-resistant OS cells is still poorly defined. In this paper, a significant decrease in miR-494-3 expression was observed in MTX-resistant OS tissues and cells, implying that miR-494-3p had an essential role in MTX resistance.

Interestingly, an online software starBase showed that miR-494-3p had putative binding sites with TGM2. Subsequently, this prediction was verified by dual-luciferase reporter assay. TGM2, a cross-linking enzyme, has been demonstrated to be tightly associated with chemosensitivity in diverse cancers [38, 39]. Moreover, previous study revealed that TGM2 expression was enhanced in cisplatin-resistant OS cells and its deficiency elevated the chemosensitivity of osteosarcoma to cisplatin [23]. Therefore, we wondered whether TGM2 participated in MTX resistance of OS. In our study, the protein expression of TGM2 was increased in MTX-resistant OS cells, and circ_0081001 served as a molecular sponge of miR-494-3p to modulate TGM2 expression. Furthermore, we found that miR-494-3p interference or TGM2 upregulation could abolish the repressive effect of circ_0081001 silence on MTX resistance in OS cells. Besides, deficiency of circ_0081001 also enhanced MTX sensitivity via upregulation of miR-494-3p and downregulation of TGM2 in vivo. All these data indicated that circ_0081001 exerted its function via the miR-494-3p/TGM2 axis. EURAMOS-1 (European and American Osteosarcoma Study Group), evaluating a multidrug chemotherapy regimen, could not contribute to an improved survival of osteosarcoma patients [40]. Therefore, circRNA-targeted therapy might be a promising treatment for OS.

In conclusion, our research identified the involvement of circ_0081001 in MTX resistance of OS cells. Knockdown of circ_0081001 enhanced MTX sensitivity in OS cells via regulating miR-494-3p and TGM2 expression. Hence, our study might contribute to a better understanding of the regulatory mechanism of MTX resistance in OS, offering a promising circRNA-targeted therapy for OS.

## Data Availability

All data generated or analyzed during this study are included in this published article.
